# Renin angiotensin system and gender differences in dopaminergic degeneration

**DOI:** 10.1186/1750-1326-6-58

**Published:** 2011-08-16

**Authors:** Ana I Rodriguez-Perez, Rita Valenzuela, Belen Joglar, Pablo Garrido-Gil, Maria J Guerra, Jose L Labandeira-Garcia

**Affiliations:** 1 Department of Morphological Sciences, Networking Research Center on Neurodegenerative Diseases (CIBERNED), University of Santiago de Compostela, Santiago de Compostela, E-15782 Spain

**Keywords:** angiotensin, estrogen, menopause, NADPH-oxidase complex, neurodegeneration, oxidative stress, Parkinson, sex differences

## Abstract

**Background:**

There are sex differences in dopaminergic degeneration. Men are approximately two times as likely as premenopausal women of the same age to develop Parkinson's disease (PD). It has been shown that the local renin angiotensin system (RAS) plays a prominent role in sex differences in the development of chronic renal and cardiovascular diseases, and there is a local RAS in the substantia nigra and dopaminergic cell loss is enhanced by angiotensin via type 1 (AT1) receptors.

**Results:**

In the present study, we observed that intrastriatal injection of 6-hydroxydopamine induced a marked loss of dopaminergic neurons in the substantia nigra of male rats, which was significantly higher than the loss induced in ovariectomized female rats given estrogen implants (i.e. rats with estrogen). However, the loss of dopaminergic neurons was significantly lower in male rats treated with the AT1 antagonist candesartan, and similar to that observed in female rats with estrogen. The involvement of the RAS in gender differences in dopaminergic degeneration was confirmed with AT1a-null mice lesioned with the dopaminergic neurotoxin MPTP. Significantly higher expression of AT1 receptors, angiotensin converting enzyme activity, and NADPH-oxidase complex activity, and much lower levels of AT2 receptors were observed in male rats than in female rats with estrogen.

**Conclusions:**

The results suggest that brain RAS plays a major role in the increased risk of developing PD in men, and that manipulation of brain RAS may be an efficient approach for neuroprotective treatment of PD in men, without the feminizing effects of estrogen.

## Background

There are sex differences in dopaminergic (DA) degeneration, as observed in animal models as well as clinical and epidemiological reports on Parkinson's disease (PD). The higher risk of developing PD in men than in premenopausal women of the same age is well-established; men are approximately two times as likely as women to develop the disease [1-3]. However, the mechanisms responsible for this difference have not been clarified [4]. It has been shown that the renin angiotensin system (RAS) plays a prominent role in sex differences in the development of chronic renal and cardiovascular diseases. The peptide angiotensin II (AII), via type 1 (AT1) receptors is one of the most important known inflammation and oxidative stress inducers, and produces reactive oxygen species (ROS) by activation of the NADPH-oxidase complex [5-7], which is the most important intracellular source of ROS apart from mitochondria [8,9]. Interestingly, RAS activity is higher in kidneys and cardiovascular tissues from males than in the same tissues from females [10-13], and males and females respond differently to stimulation and inhibition of RAS [14,15]. Furthermore, it has been shown that expression of vascular and renal AT1 receptors, as well as the balance between AT1 and AT2 receptors may be modulated by sex hormones, and a major role for RAS in the gender differences in the development of chronic renal and cardiovascular diseases has been proposed [10-12]. Several studies have revealed that estrogen-mediated down-regulation of the renin-angiotensin system (RAS) mediates beneficial effects of estrogen (E2) in several tissues [16-18]. Furthermore, there is substantial evidence that androgens may upregulate RAS activity and therefore amplify gender-related differences [10,19,20].

The brain possesses a local RAS [21,22]. We have previously shown that there is a local RAS in the substantia nigra and that DA cell loss is enhanced by AII via AT1 receptors and activation of the microglial NADPH-oxidase complex in several animal models of PD [23-25]. However, it is not known if there are differences between males and females in RAS activity in the substantia nigra, which may also be involved in the higher risk of developing PD in men than in premenopausal women. In the present study, we compared the effects of the DA neurotoxin 6-hydroxydopamine (6-OHDA) on DA neuron degeneration in male rats and rats with high stable levels of E2 (i.e. similar to proestrus), and investigated the nigral RAS in both groups of animals. Several studies have shown that the risk of developing several E2-related diseases varies with the menstrual cycle in women [26,27]. However, normal rat females have a 4-day estrous cycle, with a very short proestrus period (i.e. only 12 hours with high levels of E2). It is therefore expected that most of these rats will have low levels of E2 when killed, and during most of the 6-OHDA lesion period (two weeks), and are thus not suitable for comparison with male rats as regards understanding gender differences in vulnerability to neurotoxins in humans. Finally, the involvement of RAS in the observed gender differences in DA neuron susceptibility to the neurotoxin was confirmed by inhibition of AT1 receptors with the AT1 receptor antagonist candesartan in 6-OHDA treated male rats, and by a second experimental approach using AT1 deficient mice lesioned with the DA neurotoxin MPTP (1-methyl-4-phenyl-1,2,3,6-tetrahydropyridine).

## Results

**Figure 1:**
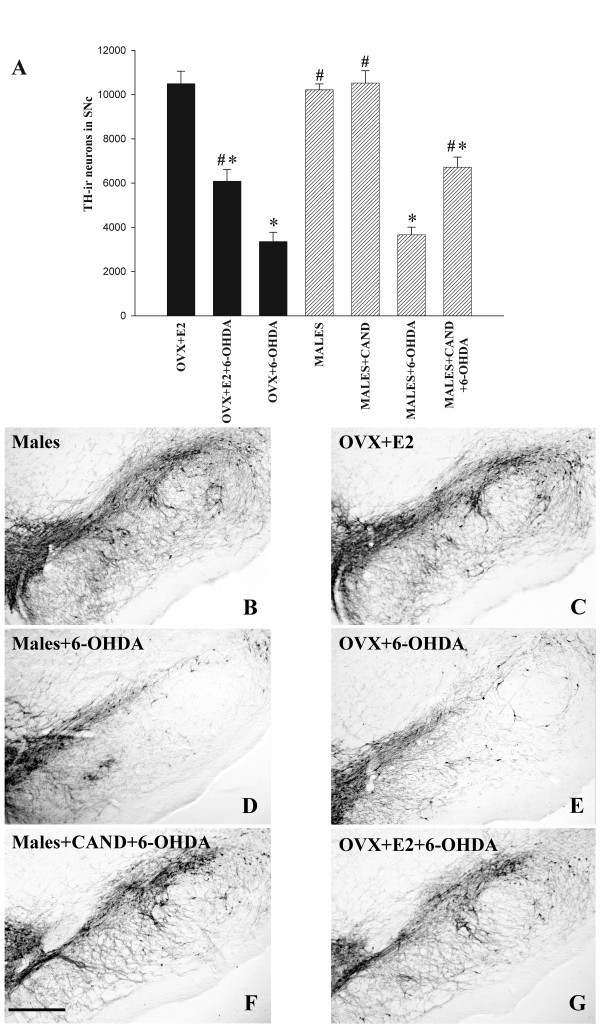
**Dopaminergic neurons in the substantia nigra compacta (SNc) of rats**. (A): Dopaminergic (TH-ir) neurons were counted in the SNc two weeks after intrastriatal injection of vehicle (i.e. controls; males and ovx+E2 females) or 6-OHDA in the different experimental groups. The dopaminergic neurons were quantified as the total number of TH-ir neurons in the SNc. Data are means ± SEM. *p < 0.05 relative to the corresponding saline-treated group (males or ovx+E2 females), ^#^p < 0.05 relative to the group treated with 6-OHDA alone (i.e. males+6OHDA and ovx+6-OHDA). One-way ANOVA and Bonferroni post-hoc test. TH-ir, tyrosine hydroxylase immunoreactive. Representative photomicrographs of TH-ir neurons in non lesioned (i.e. control) males (B), females with estrogen (ovx+E2; C), 6-OHDA lesioned males (D) and 6-OHDA lesioned females without (E) and with estrogen (G), as well as male rats lesioned with 6-OHDA and treated with the AT1 antagonist candesartan (cand; F). Scale bar: 500 μm.

Intrastriatal injection of 6-OHDA induced a similar and marked loss of DA neurons in the substantia nigra of male rats and female rats without estrogen (i.e. ovx rats), which was significantly higher than that induced by 6-OHDA in female rats with estrogen (ovx + E2). Interestingly, however, the loss of DA neurons was significantly lower in male rats treated with the AT1 antagonist candesartan, and similar to that observed in female rats with estrogen. The present results therefore show that candesartan induced neuroprotection in male rats against 6-OHDA similar to that induced by estrogen in female rats. There was no significant difference in the number of TH-ir neurons between control males injected with vehicle and male rats treated with candesartan alone (Figure 1). In order to confirm that 6-OHDA induced cell death and not only phenotypic down-regulation of tyrosine hydroxylase (TH) activity, series of sections through the entire substantia nigra of control rats and rats treated with 6-OHDA were counterstained with cresyl violet, and the total number of neurons in the SNc was estimated. The number of neurons in unlesioned females (ovx+E2; 12688 ± 472) and males (13067 ± 540) was much higher than in 6-OHDA-lesioned ovx females (4318 ± 441) or males (4856 ± 317), which was significantly lower than in females with estrogen (ovx+E2+6-OHDA; 7732 ± 346) or males treated with candesartan (males+6-OHDA+cande; 8421 ± 417). As expected, the number of Nissl-stained neurons counted in Cresyl-violet stained sections was slightly higher than that of TH-immunoreactive (TH-ir) neurons since some non-dopaminergic neurons located in the area of the SNc were also included.

**Figure 2:**
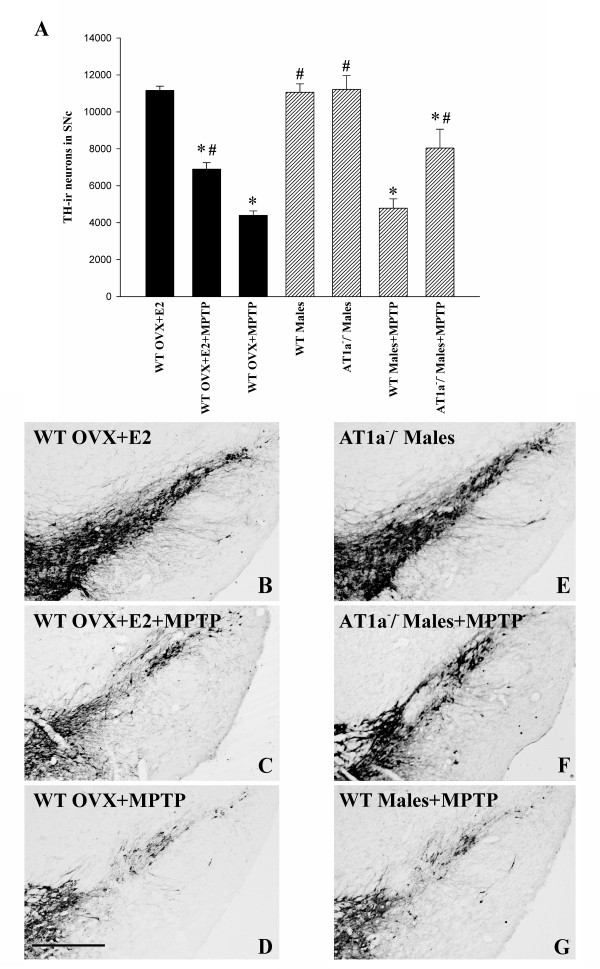
**Dopaminergic neurons in the substantia nigra compacta (SNc) of mice**. (A): Dopaminergic (TH-ir) neurons were counted in the SNc one week after the last intraperitoneal injection of saline (i.e. controls; WT and AT1a-null males and WT ovx+E2 females) or MPTP in the different experimental groups. The dopaminergic neurons were quantified as the total number of TH-ir neurons in the SNc. Data are means ± SEM. *p < 0.05 relative to the corresponding saline-treated group (males or ovx+E2 females), ^#^p < 0.05 relative to the group treated with MPTP alone (i.e. WT males+MPTP and WT ovx+MPTP). One-way ANOVA and Bonferroni post-hoc test. (B-G): representative photomicrographs of TH-ir neurons in non lesioned WT females with estrogen (ovx+E2; B), MPTP lesioned WT females with (C) and without (D) estrogen, as well as non-lesioned AT1a-null male mice (AT1a^-/-^; E), AT1a-null male mice lesioned with MPTP (F), and MPTP lesioned WT males (G). AT1^-/-^, AT1a-null mice; TH-ir, tyrosine hydroxylase immunoreactive; WT, wild type mice. Scale bar: 100 μm.

The involvement of the RAS with regard to gender differences in DA degeneration was confirmed by using a second experimental approach in which AT1a-null mice were lesioned with the DA neurotoxin MPTP. Administration of MPTP induced a similar and marked loss of DA neurons in the substantia nigra of wild type (WT) male mice and WT female mice without estrogen (i.e. ovx WT mice), and was significantly higher than that induced by MPTP in female WT mice with estrogen (ovx WT+E2). However, the loss of DA neurons was significantly lower in male AT1a-null mice, and similar to that observed in female WT mice with estrogen (Figure 2). In order to confirm that MPTP induces cell death and not only phenotypic down-regulation of TH activity, series of sections through the entire substantia nigra of different groups of mice were counterstained with cresyl violet, and the total number of neurons in the SNc was estimated. The number of neurons in unlesioned females (ovx+E2; 14760 ± 876), WT males and AT1a-null mice (14209 ± 947 and 13976 ± 756, respectively) was much higher than in MPTP-lesioned ovx females (5815 ± 698) or WT males (6070 ± 598), which was significantly lower than in females with estrogen (ovx+E2+MPTP; 8911 ± 908) or AT1a-null males (AT1^-/^-+ MPTP; 10553 ± 681), confirming that MPTP induced cell death and not TH-downregulation in the present experimental conditions.

**Figure 3:**
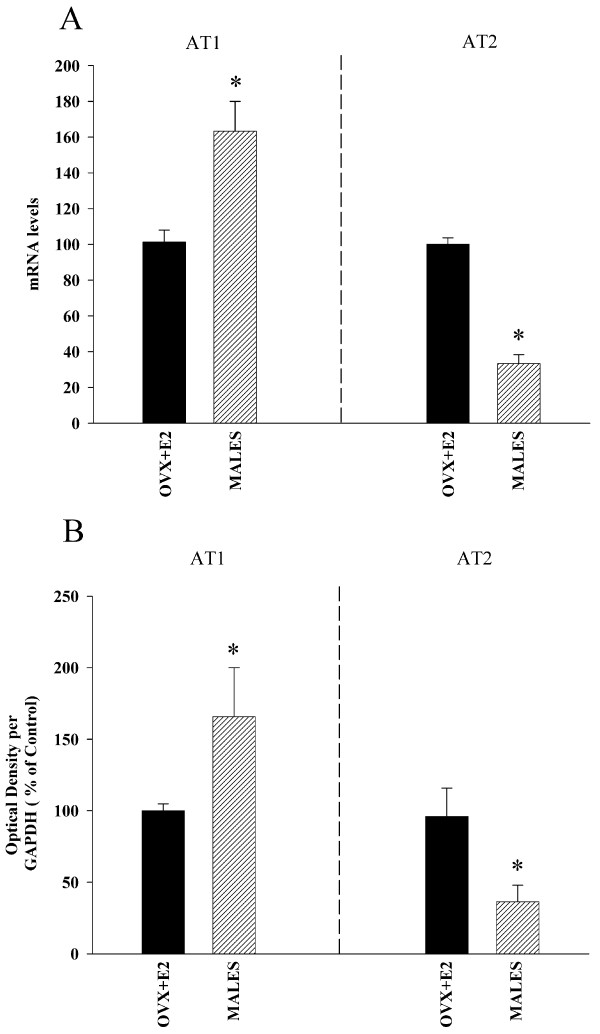
**Angiotensin receptor expression in male and female rats**. Real-time quantitative RT-PCR (A) and Western blot (WB; B) analysis of AT1, AT2 receptor expression in male rats as compared with female rats with estrogen (ovx+E2). Protein expression was obtained relative to the GAPDH band value and the expression of each gene was obtained relative to the housekeeping transcripts (β-Actin). The results were then normalized to ovx+E2 values (100%). Data are mean values ± SEM. *p < 0.05 (Student's *t *test).

**Figure 4:**
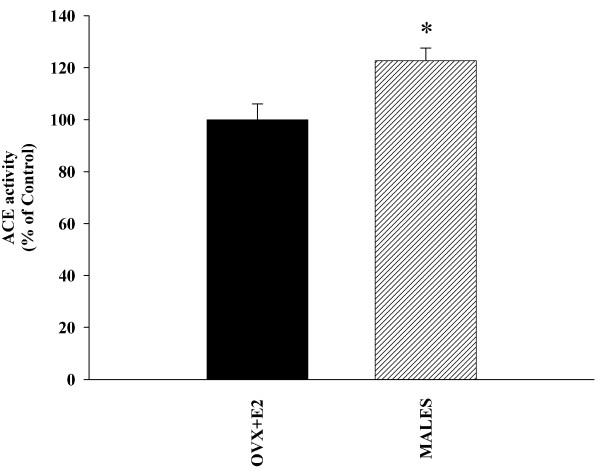
**Activity of the angiotensin converting enzyme (ACE) in male and female rats**. ACE activity in the ventral mesencephalon of males and female rats with estrogen (ovx +E2). ACE activity was significantly higher in male rats. Data were obtained as nmoles of his-leu produced per milligram of protein per minute and the results were then normalized to the values for ovx+E2 females (100%). Data are means ± SEM. *p < 0.05 (Student's *t *test).

**Figure 5:**
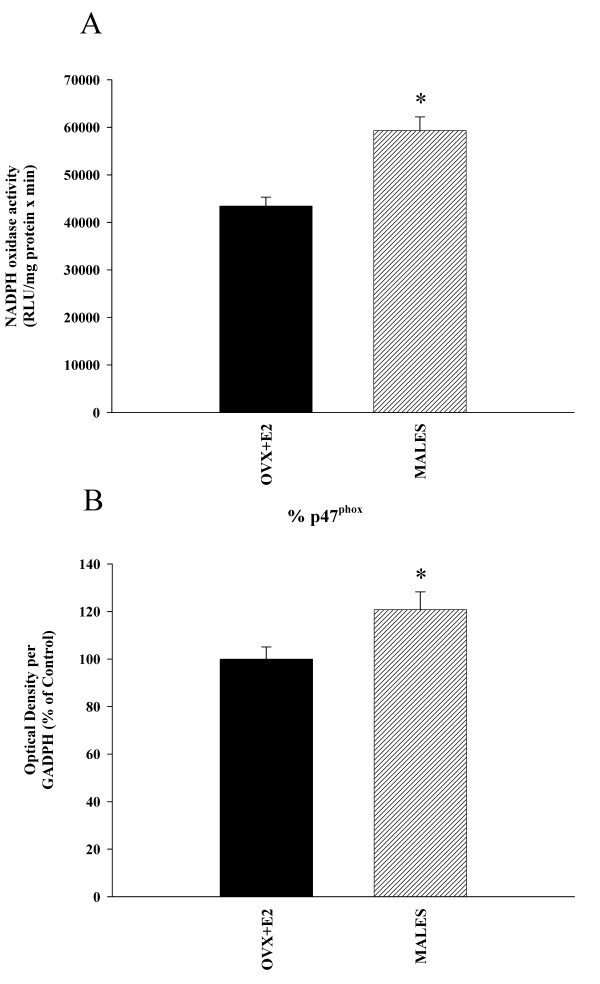
**Activity of the NADPH oxidase complex in male and female rats**. Lucigenin-enhanced chemiluminescence (**A**) and western blot (**B**) analysis of the NADPH complex activation in the ventral mesencephalon of males and female rats with estrogen (ovx +E2). Chemiluminescence analysis revealed significantly higher NADPH activity in males (**A**). Western blot analysis revealed that males had significantly higher expression of NADPH oxidase subunit p47^phox ^(**B**). Data, expressed as relative light units (RLU/min/mg protein; A) and p47^phox ^protein expression (B) was obtained relative to the GAPDH band value and then normalized to ovx+E2 values (100%). Data are means ± SEM. *p < 0.05 (Student's *t *test).

**Figure 6:**
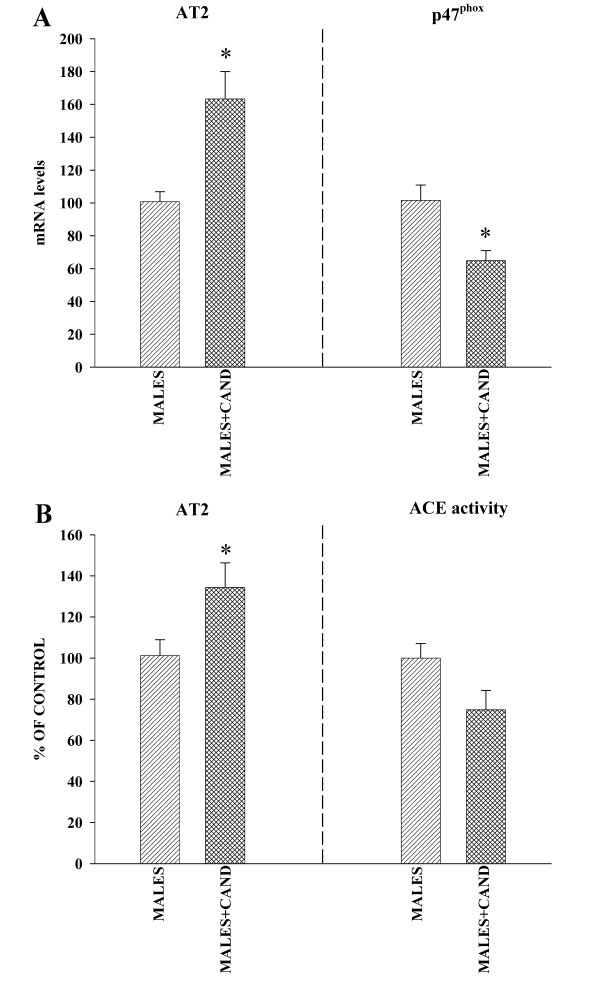
**Analysis of changes induced by treatment candesartan in male rats**. Real-time quantitative RT-PCR (A) and Western blot (WB; B) analysis, and activity of the angiotensin converting enzyme (ACE; B) in male rats treated with candesartan. Blockage of AT1 receptors induced a significant increase in the expression of AT2 receptor and significant decrease in the expression of NADPH oxidase subunit p47^phox ^in comparison with the corresponding untreated male rats; the activity of the angiotensin converting enzyme (ACE) was not significantly lower than in untreated rats. Protein expression was obtained relative to the GAPDH band value and the expression of each gene was obtained relative to the housekeeping transcripts (β-Actin). The results were then normalized to the values for control male rats (100%). Data are means ± SEM; *p < 0.05 (Student's *t *test).

Real time RT-PCR analysis revealed significantly higher expression of AT1 receptor mRNA (around 160%) and much lower levels of AT2 mRNA (about 70% reduction) in male rats than in female rats with estrogen (Figure 3A). Similarly, WB studies revealed a significantly higher expression of AT1 receptors in male rats than in female rats with estrogen, and the expression of AT2 receptors was significantly lower (about 65% reduction) in males (Figure 3B). ACE activity was significantly lower in female rats with estrogen than in males, which indicates increased AII production in males (Figure 4). In accordance with this, male rats showed significantly higher NADPH complex activity than female rats (Figure 5A). In previous studies, we observed the presence of several NADPH complex subunits, including p47^phox^, in DA neurons and glial cells (microglia and astrocytes; see references 24 and 25 for details). The increased NADPH complex activation was confirmed by the increase in the expression of the NADPH-oxidase subunit p47^phox ^in males (Figure 5B). Expression of the NADPH-oxidase cytosolic subunit p47^phox ^is an indicator of the level of activation of the NADPH-oxidase complex. The NADPH-oxidase complex is composed of membrane-bound subunits and cytosolic subunits such as p47^phox^, which is considered a key subunit for NADPH-oxidase activation [28]. Translocation of cytosolic subunits to the membrane, which leads to generation of ROS is a necessary step for NADPH-oxidase activation. The level of the NADPH-oxidase subunit p47^phox ^expression is correlated with NADPH-oxidase activity and NADPH-derived superoxide formation [7,29]. Finally, we confirmed that inhibition of AT1 receptors with candesartan induced a decrease in RAS activity in male rats. Candesartan induced a significant increase in the expression of AT2 receptors, as well as a decrease in the expression of the NADPH-oxidase subunit p47^phox^. Changes in ACE activity were statistically not significant (Figure 6).

**Figure 7:**
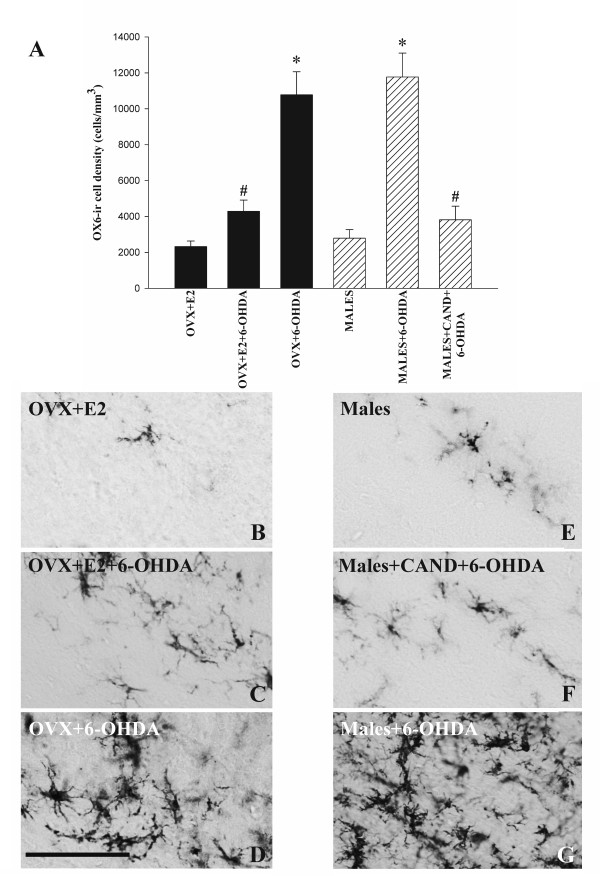
**Activated microglial cells in the substantia nigra compacta (SNc)**. (A): density of OX6-positive cells in the SNc of female and male rats of different experimental groups treated with vehicle or 6-OHDA. The microglial cells were quantified as the number cells per mm^3^, and the data are means ± SEM. *p < 0.05 compared with rats treated with vehicle, ^#^p < 0.05 compared with rats treated with 6-OHDA alone (One-way ANOVA and Bonferroni post-hoc test). (B-G): photomicrographs showing activated microglial cells at central levels of the substantia nigra in different experimental groups. Microglial activation was significantly higher in male rats and ovx female rats treated with 6-OHDA alone (D, G). Scale bar: 100 μm.

In previous studies, we observed AT1 and AT2 receptors in DA neurons and glial cells (astrocytes and microglia), and that AII induces microglial activation and DA cell death, via AT1 receptors and activation of the NADPH complex (see references 24 and 25 for details), which may explain the greater effect of DA neurotoxins observed in males than in female rats with estrogen. In order to confirm that the different response to 6-OHDA was associated with inhibition of the 6-OHDA-induced microglial response, we analyzed the expression of OX6 in the substantia nigra, as a marker for activated microglia. Control rats (i.e. ovx+E2 females and males injected with vehicle) showed minimal microglial activation. In male rats and ovx female rats injected with 6-OHDA, microglial activation was much higher than in controls. However, 6-OHDA-induced microglial activation was significantly lower in male rats treated with candesartan (males+cand+6-OHDA) and in female rats with estrogen (ovx+E2+6-OHDA; Figure 7).

## Discussion

PD is usually considered a multifactorial process in which low and apparently non-toxic doses of several pathogenic factors can act synergistically to cross the threshold of the DA cell degeneration [30] and oxidative stress and inflammation play major roles in the synergistic process [31-33]. Factors that increase the oxidative and inflammatory state of DA neurons may therefore increase the risk of developing PD. In the present study we observed higher RAS activity in male rats and mice than in females with stable high levels of E2 (i.e. similar to proestrus); the observed upregulation of RAS activity may contribute to increased DA cell vulnerability in males. Male rats showed increased ACE activity, increased AT1 expression and decreased AT2 expression, as well as increased NADPH activity and p47^phox ^expression. It is known that AII acts via AT1 receptors to induce inflammatory responses and to release high levels of ROS mainly by activation of the NADPH complex in vascular degenerative disease and other diseases mediated by oxidative stress and chronic inflammation [7,34]. In the nigrostriatal system of animal models of PD (i.e. rats lesioned with 6-OHDA and mice lesioned with MPTP), we have previously shown that brain AII induces activation of the NADPH complex via AT1 receptors, leading to increased neuroinflammation, oxidative stress and DA cell death [23-25].

The increased ACE activity in male rats leads to increased AII production. The observed upregulation of AT1 receptors in male rats may also contribute to NADPH activation and increased DA cell vulnerability. This is supported by the present experiments, in which we have observed that the enhanced susceptibility of DA neurons was significantly decreased by AT1 receptor inhibition with candesartan or deletion of AT1a receptors. Furthermore, the decrease in DA neuron susceptibility to MPTP observed in AT1a-null mice shows that the neuroprotective effect is related to the blockage of AT1 receptors and not to any other possible pharmacological effect of candesartan. It is particularly interesting that male rats also showed significantly fewer AT2 receptors than female rats, which may further enhance DA cell loss. AT1 and AT2 receptors have opposing effects and AT2 receptors counterbalance the deleterious effect of AT1 receptor stimulation, so that functional interactions between the two receptor subtypes and their specific distribution determines the AII-induced effects [35], which in the case of male rats resulted in a pro-oxidative state as suggested by increased NADPH activity and p47^phox ^expression. Interestingly, treatment with the AT1 antagonist candesartan induced a significant increase in AT2 receptor expression in males, which may also contribute to the decrease in NADPH activity and the neuroprotective effects of candesartan. An increase in AT2 expression after treatment with AT1 antagonists has also been observed in previous studies [36,37]. Furthermore, AT1 receptor blockage may also lead to preferential activation of the unopposed and upregulated AT2 receptors by similar levels of AII, as no significant change in ACE activity was observed.

We have previously shown NADPH expression in dopaminergic neurons and microglial cells. However, it appears that the AII-induced increase in microglial NADPH-oxidase activity plays a major role. It is known that in non-inflammatory cells, such as neurons, the NADPH complex produces only low rates of ROS for signaling function. In inflammatory cells such as microglia, NADPH activation produces high concentrations of ROS that are released extracellularly to kill invading microorganisms or cells [8,9]. In accordance with this, we observed that AII was not able to increase DA neuron death in the absence of microglial cells [24,25]. The present data showing differences in 6-OHDA-induced microglial activation in male and female rats confirmed the involvement of the microglial response in the sexual dimorphism of 6-OHDA neurotoxicity, and that inhibition of RAS activity with candesartan inhibits the enhanced microglial response in male rats.

The results of the present study (i.e. upregulation of RAS activity in male rats) suggest that RAS plays a major role in the higher risk in men than in premenopausal women of developing PD. It is known that neuroinflammation and microglial activation play a major role in the progression of PD [32,33,38]. AII via AT1 receptors is one of the most important inflammation and oxidative stress inducers, and a number of recent studies suggest that anti-inflammatory actions are at the core of estrogen-induced protective actions on different tissues [16,39,40]. Similarly, several studies have also shown that modulation of the glial neuroinflammatory response by estrogen is involved in the neuroprotective effects exerted by this hormone [41,42], and the present results suggest that the brain RAS is also involved. The exact mechanism of interaction between E2 and RAS has not been clarified. It has also been suggested that E2 may inhibit the effects of AII by inhibiting NADPH-derived ROS production [43], and that E2 may modulate Rho kinase signaling or other downstream pathways involved in RAS signaling [44].

In addition to the lack of estrogen, additional factors may increase RAS activity and the risk of developing PD in men. Several studies have shown that testosterone may upregulate RAS activity and therefore amplify gender-related differences [10,19,20]. It has been suggested that testosterone may modulate downstream pathways involved in RAS signaling such as Rho kinase [45]. Sex chromosomal complement may also influence AT2 receptor expression since the AT2 gene is located on the X chromosome [46]. Thus, it has been reported that AT2 receptors are absent from the kidneys of adult male rats [47], or are detected at lower levels than in females [48]. However, some studies have observed that sex differences in some RAS components in the kidney are E2-dependent and sex chromosome-independent [13]. As commented above, AT2 receptor stimulation acts in opposition to and in equilibrium with AT1 receptors, and exerts anti-inflammatory and antiproliferative effects in several tissues [35,49]. Furthermore, additional factors can further increase RAS activity and DA vulnerability in males. Firstly, aging is a particularly important factor, since advancing age itself is one of the most significant risk factors for the development of neurodegenerative diseases such as PD, and we have shown that RAS activity and oxidative stress and inflammation markers are significantly higher in aged male rats than in young male rats [50]. Secondly, we recently observed that an extensive DA denervation (i.e. 6-OHDA lesion) or functional DA depletion induced an increase in RAS activity and NADPH activity in the substantia nigra [51]. Similarly, several recent studies have revealed a counterregulatory interaction between the dopaminergic and RAS systems in several peripheral tissues, which plays a major role in degenerative changes in renal and cardiovascular systems [52-54]. This suggests that in males, an initial higher susceptibility to DA cell death and greater loss of DA terminals and DA depletion may lead to further increase in RAS activity and contribute to increased progression of PD in males.

## Conclusion

The results suggest that brain RAS plays a major role in the increased risk of developing PD in men, and that manipulation of the brain RAS may be an efficient approach for neuroprotective or coadjutant treatment of PD in men since estrogen-like effects can be obtained without the feminizing effects of estrogen.

## Methods

### Experimental design

Young adult female and male Sprague-Dawley rats (ten weeks old at the beginning of the experiments; n = 79) and male C57BL-6 mice weighing 20-25 g (i.e. 7 weeks old; n = 43) were used. Mice were wild type (Charles River, France) or homozygous mice deficient for AT1a (the major mouse AT1 isoform and the closest murine homolog to the single human AT1; [55]; Jackson Laboratory, Bar Harbor, ME, USA). All experiments were carried out in accordance with the "Principles of laboratory animal care" (NIH publication No. 86-23, revised 1985) and approved by the corresponding committee at the University of Santiago de Compostela. The animals were anesthetized with ketamine/xylazine anesthesia prior to surgery, and were fed with 2014S Teklad Rodent Maintenance Diet (Harlan Laboratories) to minimize the occurrence of natural phytoestrogens. The animals were divided into 3 groups. Rats or mice in group A were females, which were ovariectomized (ovx) and given empty implants (see below; n = 4 rats and 6 mice). Rats or mice in group B were females, which were ovariectomized and given implants containing 17β-estradiol (ovx + E2; see below; n = 27 rats and 12 mice). Group C were male rats or mice (wild type or AT1a-null mice) given empty implants (n = 48 rats and 25 mice).

In the first series of experiments female or male rats (n = 35; 15 females and 20 males) and female or male mice (wild type and AT1a-null mice; n = 43; 15 AT1a-null males, 10 WT males, and 18 WT females) were used to determine the effect of the presence of E2 or the AT1 receptor antagonist candesartan or AT1 deletion (i.e. inhibition of RAS activity by AT1 blockage) on the DA degeneration induced by the neurotoxin 6-OHDA in rats and MPTP in mice in comparison with the corresponding controls injected with vehicle. A second series of experiments was carried out to investigate the levels of RAS components and markers of NADPH-oxidase activity in male rats and female rats with estrogen (ovx+E2; n = 44; 28 males and 16 females; 2 nigral areas per rat). Rats and mice in the first series of experiments were injected intrastriatally with 6-OHDA (rats) or intraperitoneally with MPTP (mice) or vehicle (controls), then killed for immunohistochemical studies (i.e., quantification of dopaminergic cell death and activated microglia), as described below. Rats in the second series of experiments were killed by decapitation three weeks after ovariectomy and/or treatment with implants. The brains were rapidly removed and coronal slices of the mesencephalon were cut with a tissue chopper set to 1 mm. To obtain substantia nigra compacta (SNc), the individual 1 mm tissue sections were dissected on a pre-cooled glass plate under a stereoscopic microscope (Leica M220). Both SNc was dissected according to Paxinos and Watson [56], frozen on dry ice, and stored separately (two SNc per rat) at -80°C until processed. 75% of the nigras were used for expression of AT1 and AT2 receptors and expression of the NADPH-oxidase cytosolic subunit p47^phox ^by Western Blot (WB) and RT-PCR studies, or Angiotensin converting enzyme (ACE) activity; 25% of the nigras were processed for NADPH oxidase activity by lucigenin-enhanced chemiluminescence (see below).

### Estrogen and Candesartan administration

Female rats or mice (groups A and B) were bilaterally ovariectomized through a dorsal incision and received Silastic implants placed subcutaneously in the midscapular region [57,58]. Rats received a single silastic implant prepared with Silastic^® ^tubing (1.47 mm ID × 1.95 mm OD, Dow Corning 508-006; VWR Scientific, Bridgeport, NJ), as described by Febo et al. [58]. Briefly, 5-mm-long sections of tubing were sealed at one end with Silastic silicone sealant (Dow Corning 732; VWR) and left to dry for 30 min. The implants were then either filled with crystalline 17-β- estradiol (17-β- estradiol benzoate; Sigma-Aldrich; group B) or were left empty (groups A and C); the open end was then sealed in the same way as the other end. Implants were air-dried and incubated in sterile saline for at least 12-16 h to allow the initial surge of high estradiol levels to be released before use. It has been observed that such implants achieve stable levels of plasma estradiol over 30 d, with a release rate of 75-100 pg/ml per 24 h [58], as confirmed in our previous studies [59]. However, stable levels of E2 have also been found to persist for only 7-24 days [60]. Therefore, rats were killed 3 weeks after implantation (i.e., 2 weeks after 6-OHDA injection, see below). Mice received implants comprising a single 5-mm-long silastic tube prepared as described above and filled with 17- β- estradiol:cholesterol (1:1) or empty silastic implants (controls). This treatment provides plasma levels of 17- β- estradiol of 87 ± 9 pg/ml. (i.e., similar to proestrus in normal mice) [61].

In addition, some male rats (n = 16) received candesartan in their drinking water (Astra-Zeneca; 3 mg/kg/day) from 7 days before the empty implants were fitted until they were killed for immunohistochemistry or determination of levels of different RAS components. It has been reported that candesartan is the most effective AT1 antagonist in crossing the blood-brain barrier, and that low doses have little effect on blood pressure and block brain AII effects [62].

### Intrastriatal injection of 6-OHDA and intraperitoneal injection of MPTP

One week after receiving empty or E2 implants, some rats in the different groups (n = 35) were injected intrastriatally with 6-OHDA or vehicle. Thirty minutes prior to intrastriatal injection with 6-OHDA or vehicle, rats were treated with the selective inhibitor for the norepinephrine transporter desipramine (Sigma, 25 mg/kg i.p.) to prevent uptake of 6-OHDA by noradrenergic terminals. The rats were injected in the right striatum with 7 μg of 6-OHDA (in 3 μl of saline containing 0.2% ascorbic acid; Sigma, USA). Stereotaxic coordinates were 1 mm anterior to bregma, 3.0 mm right of midline, and 5.5 mm ventral to the dura; tooth bar at -3.3. Control animals were injected with 3 μl of sterile saline alone. Rats were killed by chloral hydrate overdose 2 weeks post-lesion (i.e. 3 weeks post-implant). Previous studies on the time course of 6-OHDA lesions have shown that the loss of TH-immunoreactive (TH-ir) neurons is complete [63] or practically complete [64] 2 weeks after administration of intrastriatal injections. Although a few DA neurons may degenerate after the two-week period, we considered it more important to kill the rats before any possible loss of E2 levels (i.e. 3 weeks after implantation and 2 weeks after 6-OHDA injection).

One week after receiving empty or E2 implants, some mice in the different groups (n = 43) were injected with MPTP (Free base, Sigma; 30 mg/kg/day in saline, intraperitoneally; for 5 days; n = 24) or intraperitoneal vehicle (n = 19). The mice were killed by chloral hydrate overdose one week after treatment with MPTP or vehicle (i.e. when the DA lesion is complete or practically complete) and then processed for histology.

### RNA extraction and real-time quantitative RT-PCR

Total RNA from the nigral region was extracted with Trizol (Invitrogen), according to the manufacturer's instructions. Total RNA (2.5 μg) was reverse-transcribed to cDNA with dNTPs, random primers, and Moloney murine leukemia virus reverse transcriptase (M-MLV; 200U; Invitrogen). Real-time PCR was used to examine relative levels of angiotensin receptors type 1 (AT1a) and type 2 (AT2) mRNA. Experiments were performed with a real-time iCyclerTM PCR platform (BioRad). β-Actin was used as housekeeping gene and was amplified in parallel with the genes of interest. The comparative Ct method was used to examine the relative mRNA expression. The expression of each gene was obtained as relative to the housekeeping transcripts. The data were then normalized to the values of the female group (ovx+E2) of the same batch (100%) to counteract any possible variability among batches. Finally, the results were expressed as mean ± SEM. Primers sequences were as follows: for AT1a, forward 5'-TTCAACCTCTACGCCAGTGTG-3', reverse 5'-GCCAAGCCAGCCATCAGC-3'; for AT2, forward 5'-AACATCTGCTGAAGACCAATAG-3', reverse 5'-AGAAGGTCAGAACATGGAAGG-3**; **for p47^phox^, forward 5'-CCACACCTCTTGAACTTCTTC-3', reverse 5'- CTCGTAGTCAGCGATGGC -3'; for β-actin, forward 5'-TCGTGCGTGACATTAAAGAG-3', reverse 5'-TGCCACAGGATTCCATACC-3'.

### Western blot analysis (WB) and ACE activity

For WB, tissue was homogenized in RIPA buffer containing protease inhibitor cocktail (P8340, Sigma) and PMSF (P7626, Sigma). The homogenates were centrifuged and protein concentrations were determined with the Bradford protein assay. Equal amounts of protein were separated by 5-10% Bis-Tris polyacrylamide gel, and transferred to nitrocellulose membrane. The membranes were incubated overnight with primary antibodies (1:200) against AT1 receptor (sc-31181), AT2 receptor (sc-9040), and p47^phox ^(sc-7660 all from Santa Cruz Biotechnology. The HRP conjugated secondary antibodies used were Protein A (NA9120V, GE Healthcare) and Protein G (18-161, Upstate-Millipore). Immunoreactivity was detected with an Immun-Star HRP Chemiluminescent Kit (170-5044, BioRad) and imaged with a chemiluminescence detection system (Molecular Imager ChemiDoc XRS System, BioRad). Blots were stripped and reprobed for anti-GAPDH (G9545, Sigma; 1:25000) as loading control. In each animal, protein expression was measured by densitometry of the corresponding band and expressed as relative to the GAPDH band value. The data were then normalized to the values of the female-group of the same batch (100%) to counteract any possible variability among batches. Finally, the results were expressed as means ± SEM.

### ACE and NADPH oxidase activity

ACE activity in ventral mesencephalic tissue was assayed with hippuryl-L-histidyl-L-leucine (Hip-His-Leu; Sigma) as substrate, as described by Hemming et al. [65]. Fluorescence was assayed in 96-well plates in an Infinite M200 multiwell plate reader (TECAN; excitation, 355; emission, 535) and determined as nmoles of his-leu produced per milligram of protein per minute. The data were then normalized to the values of the female-group of the same batch (i.e., expressed as a percentage of the female values; 100%). NADPH oxidase activity in ventral mesencephalic tissue was measured by lucigenin-enhanced chemiluminescence with an Infinite M200 multiwell plate reader (TECAN), as described by Griendling et al. [66] and Hong et al. [67], respectively. Chemiluminescence was expressed as relative light units (RLU/min/mg protein).

### Immunohistochemistry. Dopaminergic neuron and microglia quantification

The animals used for immunohistochemistry (i.e. those injected 6-OHDA or MPTP or vehicle) were first perfused with 0.9% saline and then with cold 4% paraformaldehyde in 0.1 M phosphate buffer, pH 7.4. The brains were removed and subsequently washed and cryoprotected in the same buffer containing 20% sucrose, and finally cut into 40 μm sections on a freezing microtome. The sections were incubated for 1 h in 10% normal swine serum with 0.25% Triton X-100 in 20 mM potassium phosphate-buffered saline containing 1% bovine serum albumin (KPBS-BSA) and then incubated overnight at 4°C with antibodies anti-tyrosine hydroxylase (TH) as DA marker (mouse monoclonal anti-TH for rat sections, Sigma, 1:10 000; rabbit polyclonal antibodies to TH for mouse sections, Peel-Freez, 1:500), or anti-OX6 (a mouse monoclonal antibody directed against a monomorphic determinant of the rat major histocompatibility complex class II antigens, expressed by activated microglia but not by resting cells; 1:200; Serotec) as a marker of reactive microglia/macrophages. The sections were subsequently incubated, first for 60 min with the corresponding biotinylated secondary antibody, and then for 90 min with avidin-biotin-peroxidase complex (ABC, 1:100, Vector). Finally the labeling was revealed by treatment with 0.04% hydrogen peroxide and 0.05% 3-3'diaminobenzidine (DAB, Sigma). In all experiments the control sections, in which the primary antibody was omitted, were immunonegative for these markers.

The total number of TH-immunoreactive (TH-ir) neurons in the substantia nigra compacta was estimated by an unbiased stereological method (the optical fractionator). The stereological analysis was carried out with the Olympus CAST-Grid system (Computer Assisted Stereological Toolbox; Olympus, Denmark). Uniform randomly chosen sections through the substantia nigra (every fourth section) were analyzed for the total number of TH-ir cells by means of a stereological grid (fractionator), and the nigral volume was estimated according to Cavalieri's method [68]. To confirm that 6-OHDA induces cell death, series of sections through the entire substantia nigra of control rats and rats treated with 6-OHDA were counterstained with Cresyl violet, and the total number of neurons in the substantia nigra was estimated by the unbiased stereology method described above for TH-ir cells. Neurons were distinguished from glial cells on a morphological basis, and neurons with visible nuclei were counted as above for TH-ir neurons. The number of OX6-ir cells (i.e. reactive microglia) was estimated using the Olympus CAST-Grid system and the unbiased stereological method described above for counting TH-ir neurons. At least four sections through the central SNc of each animal were measured. The density of OX6-ir cells (cells/mm^3^) was determined by dividing the number of labeled cells by the volume that they occupied (see references 23 and 24 for details).

### Statistical analysis

All data were obtained from at least three independent experiments and were expressed as means ± SEM. Two-group comparisons were analyzed by a Student's *t *test and multiple comparisons were analyzed by one-way ANOVA followed by a post-hoc Bonferroni test. The normality of populations and homogeneity of variances were tested before each ANOVA. Differences were considered significant at p < 0.05. Statistical analyses were carried out with SigmaStat 3.0 from Jandel Scientific (San Rafael, CA, USA).

## List of abbreviations

(6-OHDA): 6-hydroxydopamine; (ACE): Angiotensin converting enzyme; (AII): Angiotensin II; (AT1): Angiotensin type 1 receptors; (AT1^-/-^): AT1a-null mice; (AT2) Angiotensin receptors type 2; (DA): Dopaminergic; (E2): Estrogen; (MPTP): 1-methyl-4-phenyl-1,2,3,6-tetrahydropyridine; (ovx): Ovariectomized; (PD): Parkinson's disease; (RAS): Renin angiotensin system; (ROS): Reactive oxygen species; (TH-ir): Tyrosine hydroxylase immunoreactive; (WB): Western Blot; (WT): wild type mice.

## Competing interests

The authors declare that they have no competing interests.

## Authors' contributions

AIR-P conducted the experiments with rats and performed quantitative PCR and statistical analysis; RV performed Western Blot and enzyme activity analysis; BJ performed the experiments with mice; PG-R performed the histological analysis, MJG and JL-G conceived the study and its design, supervised the project and edited the manuscript preparation. All authors have read and approved the manuscript.

## Authors' information

Laboratory of Neuroanatomy and Experimental Neurology, Dept. of Morphological Sciences, Faculty of Medicine, University of Santiago de Compostela. Networking Research Center on Neurodegenerative Diseases (CIBERNED, Instituto de Salud Carlos III), IDIS (Instituto de Investigaciones sanitarias de Santiago). Santiago de Compostela, Spain.
